# Correction: From colorblind to systemic racism: Emergence of a rhetorical shift in higher education discourse in response to the murder of George Floyd

**DOI:** 10.1371/journal.pone.0318947

**Published:** 2025-02-04

**Authors:** The PLOS One Staff

## Notice of Republication

This article was republished on January 24, 2025, to correct an error in the author list: Alison Frisella was erroneously displayed as Alison Frisellaa, and was listed as affiliated with Department of Electrical and Computer Engineering, Boston University, Boston, Massachusetts, United States of America instead of Lesley University, Social Sciences Department, Cambridge, Massachusetts, United States of America. The publisher apologizes for the error. Please download this article again to view the correct version. The originally published, uncorrected article and the republished, corrected articles are provided here for reference.

## Supporting information

S1 File Originally published, uncorrected article. (PDF)

S2 File Republished, corrected article. (PDF)
