# Supplementary material for: From colorblind to systemic racism: Emergence of a rhetorical shift in higher education discourse in response to the murder of George Floyd
Source: PLoS One. 2023 Aug 3;18(8):e0289545. doi: 10.1371/journal.pone.0289545 (PMC10399877; doi:10.1371/journal.pone.0289545)
Supplement: Attachment — Submitted filename: review_PLOS_one.pdf [file pone.0289545.s001.pdf]

Review of “We are saddened by the tragic events of the last...”: A topic modeling analysis of responses by Institutions of Higher Education in the United States to the murder of George Floyd

1 Decision / Summary

I advise an accept given that my questions and comments are answered. Authors exploited a novel idea of analyzing statements on a salient issue by educational institutions with a machine-learning approach. Authors analyze statements released on a white police officer’s murder of George Floyd. The statements were collected from 356 institutions of higher education and investigated using topic modeling and ERGM. From the corpus, authors identify 18 topics in 5 domains for the discourses on race.

2 Comments

1. Page 9, Lines 165-167: Is there a case of releasing multiple statements from the same institution? If so, how did you deal with such cases?
2. Page 9, Line 172: Please include a descriptive statistics of documents such as document length realsed by each university. It seems that Table 1 only has the summary statistics of the universities.
3. Page 10, Line 180: When does the dataset of College Facutal accessed? It would be helpful to include the year that the College Facutal dataset is released.
4. Page 11, Lines 212-213: Please include your reasoning of using the highest number – 42 days – not the mean value (or some ohter days) for the statements with missing time stamps.

5. Page 12, Lines 223-228: Do the documents meet assumptions for Latent Dirichlet Allocation?

6. Page 12, Lines 231-232: Please try additional ways of checking the optimal number of topics systematically such as log-likelihood or perplexity.

7. Page 13, Line 239: Add examples of tokens (e.g., tokenized verbs).

8. Page 13, Lines 258-260: Include network visualization of the bipartite graph – either the full set of topics and statements at a glance or a subset of them.

9. Pages 16-17, Lines 328-329: Include additional explanations on how you define the 'race-centric' issues and group (i.e., domain) of topics.

10. Pages 23, Lines 370. What do you mean by "invested" in a topic? Add a line or two for explanation.

11. Page 29, Lines 493-494. The region homophily has a negative value in Table 3. On the other hand (Page 30, Lines 527-529), schools tend to have the same topics if co-located in some states. Is it for 'red' and 'blue' but not 'swing' states? Please include supporting evidence.
