# Supplementary material for: From colorblind to systemic racism: Emergence of a rhetorical shift in higher education discourse in response to the murder of George Floyd
Source: PLoS One. 2023 Aug 3;18(8):e0289545. doi: 10.1371/journal.pone.0289545 (PMC10399877; doi:10.1371/journal.pone.0289545)
Supplement: Attachment — Submitted filename: review_PLOS_one_new.pdf [file pone.0289545.s003.pdf]

Review of “From Colorblind to Systemic Racism: Emergence of a Rhetorical Shift in Higher Education Discourse in Response to the Murder of George Floyd”

1 Decision

I advise an accept. Authors provided thoughtful answers to my questions and comments. I am happy that authors significantly improved the paper.

2 Minor Comments

1. In the abstract: “statements issued in May 2020” is not correct given that the maximum time window for statement issuance does not fall into a single month.
2. Page 7, Line 141 and Page 38, Line 723: May 2020 is not Summer. Please be consistent when stating the time.
3. Page 10, Line 216: Add references to prior research.
4. Page 11, Line 244: It is written that the last statement is made 41 days after while it is written as “forty-two” days in Page 14, Line 291.
5. Page 16, Line 351: How do you measure whether a topic composes a large proportion of a statement?
6. Page 35, Line 669: Why are you stating the interpretation as “potential” interpretation?
