# Supplementary material for: From colorblind to systemic racism: Emergence of a rhetorical shift in higher education discourse in response to the murder of George Floyd
Source: PLoS One. 2023 Aug 3;18(8):e0289545. doi: 10.1371/journal.pone.0289545 (PMC10399877; doi:10.1371/journal.pone.0289545)
Supplement: Attachment — Submitted filename: Response Letter Final.docx [file pone.0289545.s002.docx]

We would like to thank the reviewers for their detailed comments and suggestions. We believe we have benefitted from a diligent re-working of the paper. A significant chunk of modifications occurs in the ‘Introduction’ section. Here, we have enriched our discussion of ‘diversity,’ describing it as an orthodoxy in Institutions of Higher Education (IHEs). We have also expanded on our discussion of ‘systemic’ racism by drawing on two major theoretical frameworks – Racial Formation Theory (RFT) and Critical Race Theory (CRT). We subsequently invoke these theories in the discussion of our results with the goal of greater coherence between the Introduction and Results sections. Finally, we bring the threads together in our ‘Discussion’ section, connecting our findings to colorblindness, diversity orthodoxy, RFT, and CRT. We have also provided more details about our choice of data, in response to suggestions and questions from both reviewers. Details of the changes we have made are below.

**Reviewer 1**

1. Page 9, Lines 165-167: Is there a case of releasing multiple statements from the same institution? If so, how did you deal with such cases?

Yes, there were few instances of multiple statements released by the same institution. If an institution released multiple statements, only the first released statement was included in our sample. We had included this information in the first draft on pages 8-9, Lines 158-159. That information has been retained in the current version of the manuscript on Page 10, Lines 229-230.

2. Page 9, Line 172: Please include a descriptive statistics of documents such as document length released by each university. It seems that Table 1 only has the summary statistics of the universities.

We thank the reviewer for drawing our attention to this blind spot. We have included data on statement length (in words and sentences) as well as time of release in Table 1.

3. Page 10, Line 180: When was the dataset of College Factual accessed? It would be helpful to include the year that the College Factual dataset is released.

We accessed data from College Factual for all variables in the month of August, 2021. This information is included in the revised Manuscript on Page 13, Line 263.

4. Page 11, Lines 212-213: Please include your reasoning of using the highest number – 42 days – not the mean value (or some other days) for the statements with missing time stamps.

We had originally fit the model using mean and median days for the missing data as well as the maximum value. We failed to include that information in the original manuscript. Much like the maximum value, neither the mean nor the median resulted in statistically significant estimates. Indeed, the estimates were nearly indistinguishable regardless of the values we used in the model. We have included the following information in the revised manuscript on Page 14, Lines 291-293:

“We also tried the mean as well as median number of days in place of the maximum. The results of the estimation remained unchanged regardless of the value used for the missing data.”

5. Page 12, Lines 223-228: Do the documents meet assumptions for Latent Dirichlet Allocation?

Yes, the documents meet the assumptions for use of LDA on the corpus. We have added some information elaborating on this on Page 15, Lines 308-311.

6. Page 12, Lines 231-232: Please try additional ways of checking the optimal number of topics systematically such as log-likelihood or perplexity.

In addition to the coherence score, we also tested the log-likelihood for our models. The log-likelihood scores largely support our decision based on the coherence score and substantive reasoning. We have added the following text to the revised manuscript on Page 15, Lines 324-324:

“We also ran the log-likelihood associated with each model. Among models with at least fourteen topics, our preferred solution with eighteen topics had the lowest log likelihood. Only models with ten or twelve topics had slightly lower scores, but, as stated above, we found those solutions to be substantively inadequate.”

7. Page 13, Line 239: Add examples of tokens (e.g., tokenized verbs).

We have added examples of tokens on Page 16, Lines 328-333.

8. Page 13, Lines 258-260: Include network visualization of the bipartite graph – either the full set of topics and statements at a glance or a subset of them.

We have included a network visualization of the one-mode projection of the complete network based on the strategy described in the paper. The original two-mode network of statements and topics was not visually appealing or informative. Instead, we found a visualization based on political affiliation of states in which schools are located to be more interesting. This is included as Figure 2 and described as follows on Page 35, Lines 656-670:

“Homophily based on political affiliation is also evident from Figure 2, which shows the one-mode projection of the complete network. The visualization algorithm places sets of nodes closer together if they are more densely connected. Schools are colored by the political affiliation of the state in which institutions are located. Red nodes depict schools located in states that voted for the Republican candidate in the 2016 and 2020 U.S. Presidential election. Blue nodes, likewise, represent schools that voted for the Democratic candidate. Finally, purple nodes represent schools located in states that voted differently across the two elections. While several schools are rendered isolate because they do not share at least one topic in common (based on the 0.1111 cutoff described in the Methods section) with other schools, a large section of the network remains densely interconnected. Consistent with the ERGM estimate, the network shows several regions of clustering based on color, illustrated in encircled portions. The implication is that schools located in states that voted similarly tend to draw on similar themes in statements. Note also that blue nodes tend to be more dominant in the center of the network suggesting that schools located in ‘blue’ states tend to share more in common with others. Another potential interpretation is that blue states used themes that were more central to the corpus.”

9. Pages 16-17, Lines 328-329: Include additional explanations on how you define the ‘race-centric’ issues and group (i.e., domain) of topics.

Thank you again for bringing this and the next point to our attention. We define degree of race-centricness based on the number of tokens in top fifteen keyworks that spoke directly to what we considered to be issues focused on race. We add the following text to elaborate on the concept on Page 20, Lines 421-427:

“These are issues that we considered to be explicitly focused on race in the United States. Topics with a high concentration of such issues contained several explicitly race-related tokens in their top keywords such as ‘black,’ ‘discrimination,’ ‘systemic racism,’ and ‘equity’. Topics with low prevalence of such issues contained almost no such tokens. The top-fifteen tokens in the second topic in Domain 5, for example, contain almost no tokens that we consider to be expressly race-related issues.”

10. Pages 23, Lines 370. What do you mean by “invested" in a topic? Add a line or two for explanation.

We added the following text to elaborate on topic investedness. This appears on Page 16, Lines 347-351:

“An edge in this network denotes the proportion of the statement being composed of a given topic. We refer to this also as the degree of a statement’s ‘investedness’ in a topic. A university or college is more invested in a topic, for example, when a higher proportion of its statement is devoted to that topic.”

11. Page 29, Lines 493-494. The region homophily has a negative value in Table 3. On the other hand (Page 30, Lines 527-529), schools tend to have the same topics if co-located in some states. Is it for `red' and `blue' but not `swing' states? Please include supporting evidence.

This is an important point that we failed to adequately explain in the first version of the manuscript. The region variable is a coarse-grained division with five categories: Northeast, South, Midwest, West, and Pacific. Political affiliation, on the other hand, links two schools if they voted the same way in the two most recent presidential elections. As we explain in the manuscript, there are two likely reasons for negative regional homophily alongside positive political homophily. First, regions are composed of politically divergent states such as New York and Pennsylvania. Second, politically convergent Democrat-voting states are located at great distances – along the two coasts and the middle of the country. As such, convergence of themes based on political similarity generates a negative estimate for regional homophily. This explanation can be seen on Page 34, Lines 635-654.

**Reviewer 2**

1. The title is too long. Unless the method is novel or the authors developed the method, I don’t recommend including the method name in the subtitle. Instead, the title should focus on the main question, evidence, and implications.

We have amended the title to be more substantively focused.

2. The same problem exists for the abstract. I recommend avoiding adjectives, such as “fundamentally,” unless they are essential for the argument.

We have also amended the abstract to focus on the primary question and findings.

3. The sampling frame is vague. The authors stated that N = 356 are most PhD-granting institutions in the US. How most are they? Put differently, are their representative of the population (PhD-granting institutions)? Also, why only focus on Ph.D. granting institutions? This decision rules out more teaching-oriented institutions such as SLACs and community colleges in the population. How was such a case selection strategy justified on what grounds?

We thank the reviewer for drawing our attention to this error. An earlier draft of the paper had focused only on PhD granting institutions, and that language slipped into the previously submitted version of the manuscript in error. We have expanded considerably on our choice of data in this revised version of the manuscript. Specifically, we focused on all colleges and universities included in the ‘National Universities’ rankings produced by U.S. News and World Report (USNWR) in 2021 (N=388). We explain in some detail why USNWR is a reasonable choice for delineating a data frame. Most significantly, we are interested in the effect of prestige and rankings on theme choice and connectedness of IHEs by theme. This would not be possible if we chose schools spanning lists. Our revised text, reproduced below, appears on Page 9, Lines 202-222:

“We focused on all IHEs included in the ‘National Universities’ rankings produced by U.S. News and World Report (USNWR) in 2021 (N=388). USNWR started publishing evaluations of colleges and universities in the early eighties. Although rankings produced by the organization have received some criticism, USNWR has come to garner tremendous legitimacy as an evaluator of universities and colleges in the U.S. [44, 45]. Their evaluations are based on a variety of indicators of academic quality such as graduation rates, faculty and student resources, and admissions selectivity. UNSWR draws on the Carnegie Classification for categorizing IHEs, a widely accepted standard in the U.S., to produce several distinct types of rankings. We draw on schools ranked in the ‘National University’ category, which includes colleges and universities that offer a range of undergraduate degrees, master’s programs, as well as doctoral degrees. These schools are also at the forefront of academic research. As such, we exclude institutions focused primarily on undergraduate education such as liberal arts colleges, regional schools, and community colleges. Our primary reason for focusing on graduate-degree granting institutions is that much prior research on institutional responses has investigated such schools. Second, our goal of investigating the effect of rankings on shared themes is only feasible if institutions are ranked on the same evaluation system. Undergraduate schools, for example, are evaluated using different metrics owing to their distinctive organizational structure. Accordingly, it would be hard to reconcile and appropriately compare schools ranked across lists (such as Williams College, ranked highly in liberal arts schools, and Princeton University, ranked highly among National Universities).”

4. The description of the colorblindness approach is vague. What does it mean that colorblindness treats racism “as a historical phenomenon.” Further elaboration on it is required. What’s the relationship between colorblindness and institutional responses? Many concepts are loosely defined and connected.

We have clarified that by ‘historical phenomenon,’ we mean something that largely existed in the past. We have replaced that language in the abstract to offer more clarity.

We have also elaborated on the relationship between colorblind ideology and institutional responses in the second paragraph on the ‘Introduction’ section. Here, we describe the results from several studies that show that college students generally tend to adhere to colorblind ideologies while in college. We also elaborate on two studies that demonstrate the use of colorblind frames in institutional responses to racist incidents on campus. The amended text appears on Page 2, Lines 41-57.

5. The description of the diversity theme is inadequate. Colorblindness is an ideology, so the diversity theme conceptualization should also be grounded in ideology. Alternatives: Multiculturalism? Racial equity and equality? Calling it diversity is too vague.

Thank you for raising this. Responding to this suggestion produced a significant addition in our manuscript. Drawing on the work of Ellen Berrey, we now elaborate on the ‘diversity’ paradigm, describing it as an orthodoxy, and accounting for its emergence and institutionalization in Institutions of Higher Education. The following amended text appears on Page 3, lines 67-85.

“As distinct from an ideology, which provides a template for the organization of the world [1], an orthodoxy constitutes a set of widely shared ideas, beliefs, and practices that guide institutional discourse as well as policy, strategy, and action.

Berrey [24] argues that, over the last two decades of the twentieth century, “diversity” became a keyword in United States Institutions of Higher Educations’ (IHEs) policies and programs surrounding race. This shift occurred, in part, due to organizational pressures in a changing political, demographic, and legal climate. An early impetus can be traced to a minority opinion issued in a significant legal case challenging affirmative action admissions policies in the late seventies. This case laid the groundwork for using diversity as a rationale for race-conscious admissions and subsequent contentious lawsuits, both challenging and supporting such policies, helped codify language surrounding diversity. Thereafter, shifts in demographics of the college going population - a rise in immigrants, foreign students, women, and people of color generated greater need for strategy and rhetoric to manage heterogeneous student populations. These strategies diffused rapidly across academic institutions becoming normative and exerting pressure on others to signal their own commitment to inclusiveness. Indeed, diversity acquired so much popularity over time that it came to replace the formerly reigning buzzword, ‘multiculturalism,’ in higher education rhetoric [22].”

6. Going back to point 3, the authors' conceptualization of the population is the Institute of Higher Education (p.4). There’s a leap from this population to the data used in the manuscript. A justification is needed. Also, it's unclear what the authors mean by the sample was selected based on the ranks produced by the US News and World Report in 2021 (also, why this index is relevant here?).

Please see our response to point 3 above where we clarify how our data are linked to U.S. News and World Report Rankings. We believe that our dataset, containing a mix of colleges and universities that offer a range of undergraduate degrees, master’s programs, as well as doctoral degrees, is a reasonable, though not perfect, representation of Institutions of Higher Education in the United States. Accordingly, we believe that the IHE phrasing is appropriate.

7. The data interpretation is not well connected to the theory. The theory assumes that two prevalent ideological themes influenced how the US colleges issued statements regarding the murder of George Floyd. How do we know that’s the case? Perhaps, the underlying cause is the institution's strategic decisions based on their constituencies. The authors did this type of reasoning when they mentioned how the prevalence of mentioning perpetrators varies by the school’s locations and their partisan characteristics (p.11, p.23). In short, many interpretations in the manuscript relied on post-hoc theorization. On the minor related point, it’s surprising that the manuscript didn’t mention “Black Lives Matter” only twice and did not delve into it! Why there's no Black Lives Matter protest variable included in the estimation model?

This comment contains several points. We believe we have addressed all of them in the revised version of the manuscript.

First, we did not make adequate connections between our results and theory in the previous version of the manuscript. We have aimed to redress this issue by systematically linking our findings to colorblind ideology and diversity orthodoxy in this revised version.

Second, we have added several paragraphs to the ‘Introduction’ section elaborating on what Warikoo and de Novais (2015) call the ‘power analysis’ frame. Specifically, we argue that this framing draws on two dominant theories of race in the social sciences - Racial Formation Theory (RFT) and Critical Race Theory (CRT). We elaborate on the core tenets of each of these theories, situating them in opposition to colorblind ideology and diversity orthodoxy. Most significantly, both theories emphasize the pervasive and enduring nature of racism in the United States. In addition to colorblindness and diversity, we also draw on CRT and RFT to make sense of our findings in the ‘Results’ as well as the ‘Discussion’ sections.

Third, there is nothing in our analysis that suggests that IHEs are not acting strategically. To the contrary, drawing on existing tropes focused on diversity, equity, and inclusion, as well as newer ones focused on systemic racism, may well be purposeful action aimed at appearing to be committed to inclusiveness.

Finally, we considered how we might incorporate ‘Black Lives Matter’ into our ERGM model. We looked for data on protests in cities and towns, but found that (1) the available data were not good enough quality, failing to account for *all* occurrences of protest, for example, and (2) when we looked to create our own data, we found that protests occurred in most towns and cities, and all states, making it very difficult to conceptualize as a variable. We would like to emphasize that one of the topics in our topic modeling analysis is labeled ‘Black Lives Matter.’ We provide details about that topic, including the number of times the trigram ‘Black Lives Matter’ appears in the corpus, on Page 25, Lines 440-446.
