# Supplementary material for: From colorblind to systemic racism: Emergence of a rhetorical shift in higher education discourse in response to the murder of George Floyd
Source: PLoS One. 2023 Aug 3;18(8):e0289545. doi: 10.1371/journal.pone.0289545 (PMC10399877; doi:10.1371/journal.pone.0289545)
Supplement: Attachment — Submitted filename: Response letter Final.docx [file pone.0289545.s004.docx]

We would like to thank the reviewers once again for their detailed comments and suggestions. We believe we have benefitted from a diligent re-working of the paper. Details of the changes we have made are below.

**Reviewer 1**

1. In the abstract: “statements issued in May 2020” is not correct given that the maximum time window for statement issuance does not fall into a single month.

Thank you for pointing this out. We have fixed this error by changing “May 2020” to “the Summer of 2020”.

2. Page 7, Line 141 and Page 38, Line 723: May 2020 is not Summer. Please be consistent when stating the time.

We have also addressed this error. We are now consistent in our use of “Summer of 2020” whenever we refer to the period when statements were issued. We only use “May 2020” when we speak of the death of George Floyd.

3. Page 10, Line 216: Add references to prior research.

Done.

4. Page 11, Line 244: It is written that the last statement is made 41 days after while it is written as “forty-two” days in Page 14, Line 291.

Thank you for picking up on this. “41” was an error in the previous manuscript. We have fixed this to read “forty-two” now (Line 261).

5. Page 16, Line 351: How do you measure whether a topic composes a large proportion of a statement?

We have offered clarification for how we measure topic composition in statements. Specifically, we arrive at topic investedness from the document-topic probability matrix. In the MS (page 16, Lines 372-374), we clarify that we “deduce this from the document-topic probabilities vector or topic mixture, which shows the estimated proportion of words from a given statement that are generated from all topics.”

6. Page 35, Line 669: Why are you stating the interpretation as “potential” interpretation?

We used the term to indicate that it was one of two possible explanations. We have replaced the word with "possible" to make it clearer for the reader.

**Reviewer 3**

1. I appreciate the inclusion of RFT and CRT. However, I think they are not well integrated into the rest of the paper. I was hoping, when reading the explanation of your LDA results, that you could tie the topics more to the two theories. When reading L132-136, I was wondering how this trend would be reflected in your analysis, but I think the current Results section hasn't elaborated on this.

We thank the reviewer for this comment, which has helped improve the MS. Specifically, throughout the results section, we outline how topic domains or individual topics reflect tenets of CRT, RFT, or diversity/colorblind race frames. For example, in describing the first topic domain, we add the following text on page 25, Lines 459-463:

“In contrast to prior literature which demonstrates the proliferation of colorblindness and diversity orthodoxy in higher education rhetoric, topics in this domain, we find, resonate strongly with the tenets of Critical Race Theory, and especially the notion that systemic racism is deeply embedded within United States’ social, political, and economic institutions.”

Please refer to the full Results section for further elaboration on CRT and RFT in relation to our findings.

2. Table 1: Is it meaningful to report the mean or median of school rankings?

This is a good point. We have removed the line for school rankings from Table 1.

3. I'm also curious about the min and max of many items.

We have added the minimum and maximum of all continuous variables: statement length (Lines 262-263), undergraduate percentage black (Lines 278-279), and undergraduate percentage female (Lines 286-287) in the description of the attribute variables.

4. 3. L352: et al[.]

Done.

5. 4. Not sure if it's a formatting error: Figure 1 and Figure 2's captions and the figures are on separate pages.

The captions appear on the same pages as the figures on our end. Perhaps this is something that is changed in the translation from the word file to the online proof generated for review purposes. We can make sure to fix this issue if the paper is accepted for publication.

6. 5. L585, I wonder if it's possible to label subfigures?

The sub-figures in Figure 1 are labeled on our end (below each bar graph). Again, this might be an issue in the translation process.

7. 6. L592: May want to mention Figure 1 just for clarity?

Done.

8. 7. L593: Table 3?

Our apologies for this error. We have fixed this typo.

9. 8. L640: "in concern", not concert?

We meant “in concert” to indicate “taken together.” We have modified the language to be clearer for readers.

10. 9. I think discussions starting from L702 should be first presented in the Results section.

We have moved several lines from the Discussion section to the Results section (Lines 558-564) to address this concern. We return to this point in the Discussion section (Lines 759-766) but refer the readers to the material presented in the Results section.

11. 10. This is more of only a comment: I think the paper ends rather abruptly. I was hoping for at least a summary of findings, if possible, a bit more implications for future research.

Thank you for this comment. We have added a concluding paragraph to close out the paper.

**Reviewer 4**

1. While authors carefully collected the data, analyzed them, and made conclusions, I found one major mismatch from the authors' arguments and the data and methods. From the title, "From colorblind to systemic racism," the abstract, "our analysis reveals two striking rhetorical shifts on racial discourse," and several places in the manuscript, authors emphasized that this article reveals the transition of discourse. However, what authors analyzed is the case of George Floyd's murder happened in May 2020. Therefore, technically speaking, authors could not reveal the transition in the discourse by looking at the one-time event. I concurred with authors that this article contributes to the existing literature by revealing the significance of university's view on systematic racism. However, it does not mean the authors' data and methods found that authors found the fundamental shift in the university's view on racism. In order to show that authors should have illustrated how universities reacted to similar events before and after the case of George Floyd's murder and showed the topic distribution change over time. Given that this path might not be the feasible way to develop the paper further, I recommend to tone down the paper's argument in transition or emergence of universities' view on racism, but focus on its contribution in understanding the case.

We would like to thank the reviewer for this suggestion/comment. We wrestled with it for some time to find a way forward. Ultimately, we have made several substantial changes to the MS, which we hope addresses this important concern.

Succinctly, with these changes, we hope to clarify that the “shift” we emphasize occurs in reference to what is known based on prior investigations of higher educational institutions. This existing body of literature establishes the dominance of colorblind ideology and diversity rhetoric in both student attitudes (Lines 42-50) and institutional responses (Lines 50-57). Moreover, prior literature shows the absence of acknowledgment of systemic racism in IHEs in the United States (Lines 57-63). Our findings show a strong prevalence of this language in statements issued by IHEs in the Summer of 2020. Thus, we make sense of our findings by situating them in reference to this prior work, thereby arriving at the language of a “shifting landscape.”

This practice of situating work and locating changes on the basis of prior knowledge is quite standard practice in our fields. Nevertheless, we agree that (1) this may not be standard practice in other fields, and, hence, to readers of Plos One, and (2) we did not make it adequately clear in the previous version of the MS that the shift was in reference to prior work. Accordingly, we have made several modifications to the MS:

i. First, we have added language to the final paragraph of the Introduction section to clarify that our analysis contributes to a body of literature that has already established the prevalence of colorblind ideology and diversity rhetoric in higher education in the United States. Specifically, we say (Lines 204-209):

“While we analyze statements released at approximately one point in time, our objective is not limited to analyzing the rhetoric in that set of responses. We also aim to compare the dialogue invoked in the Summer of 2020 to findings from prior literature, which clearly shows the dominance of colorblind ideology and diversity orthodoxy in dealing with issues related to race and racism in U.S. IHEs.”

ii. We have added a modifier (Line 19) to the abstract to clarify that the shift should be interpreted in reference to prior work.
iii. We have elaborated on the second paragraph of the introduction to emphasize the prevalence of colorblindness in IHEs in the U.S (Lines 42-63).
iv. In addition to instances where we had already noted departures from prior literature in the previous round of the MS (e.g., Lines 508-510 and 512-516), we have added materials in several places in the Results section to clarify that our results point to trends that are distinct from prior findings.
v. We have done the same in the Discussion section. Significantly, every time we refer to shift in rhetoric (e.g., Lines 732-734, 742-746,764-766, 768-771, and others) we clarify that the change is only evident when we compare our findings to prior literature, which shows the dominance of colorblind ideology and diversity orthodoxy.
