# Supplementary material for: From colorblind to systemic racism: Emergence of a rhetorical shift in higher education discourse in response to the murder of George Floyd
Source: PLoS One. 2023 Aug 3;18(8):e0289545. doi: 10.1371/journal.pone.0289545 (PMC10399877; doi:10.1371/journal.pone.0289545)
Supplement: Attachment — Submitted filename: Response to reviewers.docx [file pone.0289545.s005.docx]

We would like to thank the editors once again for their detailed comments and suggestions. We believe we have benefitted from a diligent re-working of the paper. Details of the changes we have made are below.

1. There is some inconsistency in the use of terminology: colorblindness vs colorblind racism vs colorblind ideology vs colorblind racist ideology vs colorblind rhetoric. This is one example. Authors should clarify if these terms are being used interchangeably or if these terms have different meaning. A table of definitions could address this issue and include other terms: diversity, diversity orthodoxy, "interest convergence", "voice-of-color" thesis, prejudice, and other race-related terms throughout the text.

Thank you for bringing this to our attention. We have resolved this concern in several ways. First, we clarify on page 1 [Lines 32-33] that the ideology of colorblind racism is also referred more simply as colorblind ideology, colorblindness, or colorblind racism in the literature. We include some citations to support our claim.

Second, we have ensured that we use only these terms when we refer to the ideology of colorblind racism anywhere in the manuscript. To this end, we have made minor modifications across the manuscript wherever we used terms different from these. For instance, we replaced “colorblind rhetoric” in the abstract with “rhetoric consistent with colorblind racism.” Likewise, we replaced the term, “colorblind tropes” with “themes consistent with colorblind ideology.”

Third, as per your suggestion, we have created a Glossary Table included at the end of the manuscript, where we define the most significant terms related to race and methodology used in the manuscript.

2. On page 1, there is a vague and convoluted explanation of terminology from a macro perspective (i.e., institutions of higher education) whereas there tend to be more clearly defined from micro or interpersonal perspectives (e.g., colorblindness at the individual level). Given the sensitivity of DEI and race-related terms, vague definitions can do more harm than good.

We addressed this comment by providing further context on how colorblind ideology manifests in interpersonal racism. Specifically, we begin the second paragraph [Line 43] of the manuscript by stating, “In interpersonal interactions, colorblind racism manifests through the denial or minimization of the role of race in shaping an individual’s experiences or outcomes, and through assertions that any advantages gained by a social group are obtained through merit rather than privileges associated with racial identity [1-2, 5-6].”

3. Lines 46-48, seem to reference the use of microaggressions by college students today, but does not directly use the term "microaggression" which is well-defined in the literature and used indirectly to make racially bias insults in professional and institutional settings.

While we are sympathetic to the editor’s comment, we re-read all the papers cited in that paragraph again and could not find the term microaggressions used anywhere in the cited literature. We believe this is because those papers are principally concerned with attitudes rather than actions, which is where the term microaggressions tends to be central. Accordingly, we do not add a discussion on microaggressions because our paper is similarly primarily focused on institutional positionality and posturing rather than action.

4. Lines 67-68, which states that "...race is viewed as a matter of cultural difference..." needs further explanation and clarification. This should not be difficult given that 5 articles are cited. Perhaps a definition of diversity should begin this discussion. In line 726, race is referred to as "cultural identity". Is this the same as "cultural difference"?

We have amended the paragraph beginning on Line 72 by adding a definition of diversity as typically used by organizations. Specifically, we state:

“In contrast to colorblindness, diversity, the second dominant theme in race discourse in the United States today, underscores the significance of race rather than minimizing it. Generally speaking, ‘diversity’ has been used by organizations, including IHEs, to refer to heterogeneity of persons based on a myriad of social and personal differences such as race, gender, ethnicity, nationality, and disability status [19-23]. In this context, akin to ethnicity, race is framed as a valued ‘cultural identity’ and racial differences, much like ethnic ones, are viewed as a matter of *cultural* heterogeneity associated with variability in behaviors, expressions, beliefs, and practices. Racial diversity is thus seen as creating conditions for heterogeneity of interactions among community members, which, in turn, are framed as generative of instrumental benefits such as a superior social climate and creativity of thought [24].”

As we have clarified that organizational usage of diversity treats race as a cultural identity and made the connection between cultural identity and cultural difference, we have retained the description of race as a ‘cultural identity’ later in the manuscript.

5. Institutional racism and systemic racism seem to be used interchangeably. There is literature to suggest that institutional racism is a component of systemic racism. Please clarify and/or use the same terminology consistently.

We have removed all mentions of ‘institutional racism’ from the manuscript.

6. Inconsistent use of "IHE" as an abbreviation.

We have made numerous changes throughout the manuscript to use ‘IHE’ as consistently as possible. We have retained other terms when they were more accurate or when they fit the prose better.

7. In lines 156-157, what are the conclusions that the studies reach about how racism is discussed in statements?

We have clarified that the conclusions reached by these studies are discussed next by combining the paragraph that came after with the current paragraph [Lines 173-180]. We hope this makes things clearer for the reader. Specifically, we say:

“Regardless of sample, these studies come to comparable conclusions about the ways in which racism is discussed in statements. Specifically, consistent with diversity orthodoxy, researchers find the themes of ‘justice,’ ‘diversity,’ and ‘inclusion’ to be featured prominently in IHEs rhetoric. In their analysis of statements released by 56 leading United States medical schools, Kiang and Tsai [14], for example, find that 40 use the term “inclusion,” 33 use “diversity,” and 29 use “justice.” The authors also note that all institutions used some form of what they characterize as ‘hopeful’ language – rhetoric that invokes diversity as having positive instrumental value.”

8. In the results and discussion, state the domain instead of referencing the number in Table 2 to prevent the reader from having to keep referencing the table. Sometimes in the discussion the domain is named and other times it isn't. Figure 1 poses the same issue regarding domain names--they are not included and there isn't a legend for each panel.

Thank you for bringing this to our attention. We have made several changes to the Analysis and Discussion sections by replacing domain and topic numbers with their substantive names or by adding domain and topic names in parentheses.

We have offered greater clarity regarding the legends for Figure 1 in the caption for the figure. We have also added a row at the bottom of the figure with Domain names. We tried to fit the domain names into the figures, but that was not possible due to the length of the names.

9. Prejudice is used for the first time in line 750.

We now use the term prejudice several times starting with the first paragraph. However, for clarity, we have added the term to the glossary.

10. Minor suggestions: RFT and CRT are spelled out and abbreviated early in the text and later in the text (line 650) both theories are spelled out again.

We have used the acronyms consistently wherever we refer explicitly to RFT and CRT after their first usage in the manuscript. We have retained the longer forms on occasion when it flowed better with the prose or to remind readers of the meaning of the abbreviation.

11. "Tropes" is introduced into the discussion late in the text (line 806) in reference to IHEs being "invested in tropes" which is not a common use of the term in a racial context.

We have replaced the term ‘tropes’ with ‘themes.’
