# Supplementary material for: From colorblind to systemic racism: Emergence of a rhetorical shift in higher education discourse in response to the murder of George Floyd
Source: PLoS One. 2023 Aug 3;18(8):e0289545. doi: 10.1371/journal.pone.0289545 (PMC10399877; doi:10.1371/journal.pone.0289545)
Supplement: Attachment — Submitted filename: Response to reviewers.docx [file pone.0289545.s006.docx]

**Editorial Comments**

“Thank you very much for the revisions you have conducted so far. We have one final comment we would like to raise:

1. Lines 46-48, infer use of microaggressions by college students today, but does not specifically use the term "microaggression" which is well-defined in the literature and used to identify to racially bias insults in professional and institutional settings. The intent of the comment was not for the authors to include a discussion on microagressions, but to explicitly state the behavior to which they are referring in the text whether they are referring to microaggressions or other behavior. The original comment indicated that the authors did not use the terminology "microagression".”

We have addressed this comment by adding material linked directly to microaggressions. We draw on a seminal paper by Sue et al. (2007) and a more recent paper by Skinner-Dorkenoo et al. (2021) to link behavior associated with color blind racism with microinvalidations, a form of microaggression. Specifically, on pp. 1-2, lines 43-50, we now say:

“In interpersonal interactions, colorblind racism manifests through the denial or minimization of the role of race in shaping an individual’s experiences or outcomes, what Sue et al. [7] describe as microinvalidation, a form of microaggression. Microinvalidations involve denying the feelings, perceptions, observations, or realities of people of color, processes that contribute to reproducing colorblind racism by suppressing the effects of racism and making it more difficult to identify [8]. This typically occurs through assertions that any advantages or disadvantages sustained by a social group are obtained through merit or its lack, rather than privileges or disprivileges associated with racial identity [1-2, 5-6].”

We have also added a comprehensive definition of microaggressions to the glossary table, which appears at the end of the manuscript.
